# Altered genome induced immune response of iPSCs

**DOI:** 10.3389/fimmu.2026.1751499

**Published:** 2026-01-28

**Authors:** Jordi Requena Osete, Belén Álvarez Palomo, Michael J. Edel

**Affiliations:** 1 Department of Medical Genetics, Oslo University Hospital and University of Oslo, Oslo, Norway; 2 Division of Psychiatry, Haukeland University Hospital, Bergen, Norway; 3 Department of Clinical Medicine, University of Bergen, Bergen, Norway; 4 Banc de Sang i Teixits, Edifici Dr. Frederic Duran i Jordà, Barcelona, Spain; 5 Autonomous University of Barcelona, Faculty of Medicine, Unit of Medical Histology, Barcelona, Spain; 6 Discipline of Medical Sciences and Genetics, School of Biomedical Sciences, University of Western Australia, Perth, WA, Australia

**Keywords:** cell differentiation, embryonic stem cells, genetic stability, immune response, induced pluripotent stem cells

## 1 Human pluripotent stem cell genomic instability

**Figure 1:**
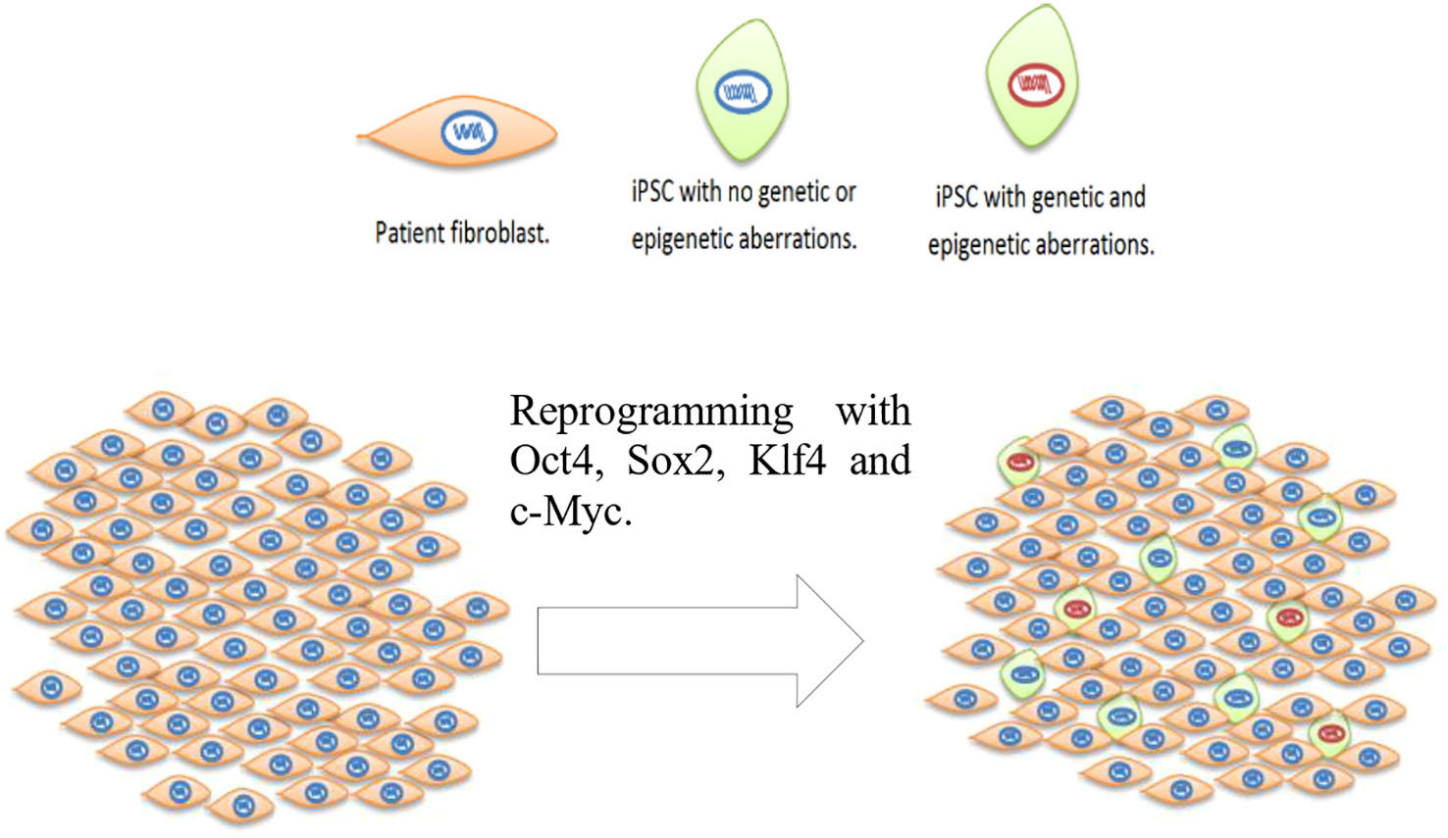
Reprogramming of patient fibroblast using a clinical therapy viable method introducing the four Yamanaka genes: Oct4, Sox2, Klf4 and c-Myc. After reprogramming the resulting population of cells contain not reprogrammed patient fibroblasts, cells reprogrammed carrying no genetic or epigenetic aberrations (green cells with blue nucleus) and cells reprogrammed carrying genetic and epigenetic defects (green cells with red nucleus). The proportion of cells carrying genetic and epigenetic aberrations is not known.

Genomic instability of human pluripotent stem cells (iPSCs) was first documented in 2004, reporting karyotypic abnormalities in human embryonic stem cells (ESCs), including trisomy of chromosome 12 (1, 2). Several reviews have been written focusing on ESCs and iPSCs (3) genomic and epigenomic instability (4–8). It is widely accepted that cell reprogramming to iPSCs can induce both genetic and epigenetic defects in iPSCs (**Figure 1**) (9–12). Analysis of non-integrative virus-free methods, such as mRNA transfection (13) and episomal vectors (14) found that the reprogramming method impacts on genomic changes in iPSCs, with mRNA reprogramming method generating less genomic instability (14, 15). Genetic alterations range from single nucleotide point mutations to whole chromosome aneuploidies (including mosaic) or sub chromosomal aberrations, including gene duplications and deletions (14, 16–18).

Genomic changes can occur during the cell reprogramming process to pluripotency, long term culture of iPSCs or during differentiation of iPSCs to various cell types, but not limited, such as neurons, cardiomyocytes and beta islets cells (19). Interestingly, late passage iPSCs were twice as likely to encumber genomic changes compared with early passage cells (33% compared with 14%), as reported in 2011 in a large-scale study of more than hundred iPSCs lines (16). This result points to the key fact that selective pressure plays an important role in favoring accumulation of genomic alterations that confers growth advantage.

### 1.1 Common genetic alterations

The most frequent chromosome duplications (whole chromosome and subchromosomal regions) in pluripotent cells are in autosome chromosomes 1, 12, 17 and sex chromosome X. Amplifications in 20q region have been detected in 34% of ESC and iPSC lines examined (14, 16, 20). Trisomy of chromosome 12 is the most recurrent abnormality in both ESC (42.6%) and iPSCs (32.9%) (14, 20). Interestingly, many chromosomal abnormalities found in ESC are also found in iPSCs. However, while chromosome 8 gains are more likely to be found in iPSCs, chromosome 17 gains are more likely to be found in ESC (14, 21, 22). High resolution single nucleotide polymorphism (SNP) analysis mapping of ESCs and iPSCs found common subchromosomal duplications in chromosome 20q, in genes conferring cell growth or survival advantage, such as BCL2L1 (20Q11.21). BCL2L1 enhances ESC survival giving therefore a selective advantage by attenuation of apoptosis; or mir1825, which has over 400 predicted targets, triggering suppression of apoptosis and cell growth enhancement (23). Importantly, recent work has demonstrated that iPSC culture introduces mutations in the BCOR gene that can affect the differentiation process, particularly to neurons and may impact other cell functions that could include the immune system, currently under investigation (24, 25).

### 1.2 Copy number variation

Gene copy number variation (CNV) by itself is not necessarily a high-risk trait. A mounting number of studies have demonstrated that somatic mosaicism of ordinary cells is a normal characteristic of the human body (26–30). However, human iPSCs have a higher number of subchromosomal CNV than ESC (17, 22, 31). Early-passage iPSCs are characterized by a huge incidence of CNV compared with parental fibroblasts. These alterations, especially copy number losses, are usually negatively selected in culture. Recently it has been described that CNV can be profiled by using a high-density DNA methylation array with the same sensitivity of SNP platforms (32). The most recurrent CNV hotspot is amplification of the gene-rich locus at the long arm 20q11.21. It is estimated to be present in approximately 14.5% of ESC and iPSC lines (16, 17, 22, 33–40). Interestingly, the alteration in this region has been reported to be culture-induced.

### 1.3 Passaging and differentiation-induced genomic aberrations

Karyotypic abnormalities frequently accumulate in ESCs and iPSCs during *in vitro* culture maintenance. Long-term culture positively selects for amplifications but negatively select for deletions (17). This phenomenon can be explained by the strong cell culture selective pressure rapidly selecting against deletions (31), favoring best adapted cells and resulting in enrichment of chromosomal trisomies and copy number gains, which contribute to the genomic variation detected in iPSCs (13, 41).

Culture-induced genomic aberrations in ESCs and iPSCs are unpredictable and variable between lines and can occur at any stage (14, 16, 17, 22, 31). Therefore, it is difficult to develop specific culture conditions to maintain homogeneous genomically stable populations and a safe passage number threshold cannot be determined.

Genomic alterations can also be selected for during differentiation of ESCs and iPSCs. For example, an abnormal subpopulation of ESCs with multiple duplications in chromosome 20, after only 5 days, was selected in a cardiac differentiation experiment to cardiomyocytes (17). Interestingly, multipotent adult stem cells also show frequent typical chromosomal abnormalities, like duplication of chromosome 19 in neural stem cells (NSCs) or a deletion of chromosome 13 in mesenchymal stem cells (MSCs) (21).

Regarding point mutations, exome sequencing has shown that 74% of mutations detected in iPSCs are generated during reprogramming, 19% pre-existed in parental fibroblasts, and only 7% are caused by *in vitro* maintenance (41). Nevertheless, selection of pre-existing subpopulations of mutant parental fibroblasts during reprogramming was found to explain this high percentage (42).

### 1.4 Epigenetic instability

Reprogramming to human iPSCs can induce epigenetic anomalies (43–46). Epigenetic alterations refer to alterations in patterns of (a) gene imprinting, (b) DNA methylations and (c) histone modification (47).

#### 1.4.1 Alterations in gene imprinting patterns

Imprinting is the epigenetic silencing found in some alleles of specific genes depending on a parent-of-origin specific manner. Typically, alterations in imprinting provide growth advantages for pluripotent cells maintained in culture because many imprinted genes are known to regulate growth during embryonic development (48). A large-scale comparison of ESC, iPSCs, somatic tissues and primary cell lines demonstrated that pluripotent cells are characterized by a high level of variation in the methylation status of a subset of imprinted genes (49). Genetic variation and instability were discovered in the imprinting status of a subset of genes in pluripotent cell lines, such as the paternally imprinted genes H19 and the maternally expressed 3 (MEG3) tumor suppressor (50).

#### 1.4.2 Alterations in DNA methylation patterns

DNA methylation in pluripotent cell lines is typical for a subset of imprinted and developmental genes, for instance the alteration in methylation of the tumor suppressor RAS association domain family member 1 (RASSF1) (51), suggesting a positive selection pressure to culture induced methylation changes. Human iPSCs have been reported to have increased levels of DNA methylations, which are aberrant and different from ESC during early passages. However, during prolonged culturing, the level of DNA methylation gradually becomes even (52). Moreover, studies with iPSC-derived neurons suggest that many DNA methylation differences between iPSCs and ESCs are largely normalized upon differentiation (53). Furthermore, it has been shown that abnormal methylation patterns in iPSCs are influenced by the choice of reprogramming factors, with different factor combinations leading to distinct patterns of methylation error (failure to demethylate *vs*. failure to methylate) (46).

#### 1.4.3 Alterations in histone modification patterns

Human iPSCs have increased levels of H3K27me3 and several studies have demonstrated differences with histone 3 trimethylations marks between ESC and iPSCs (9, 54, 55). Other studies demonstrated that lysine 9 (H3K9me3) rather than lysine 27 (H3K27me3) is highly modified (30). Lysine 4 (H3K4me3) variation patterns were found to be similar (54). In addition, such changes were also reflected at the transcript level with changes in the expression of multiple genes involved in developmental and epigenetic processes.

## 2 Immunogenic potential of autologous cell therapy

The immunogenicity of iPSC-derived cells is a subject of ongoing research. Guha et al. found that transplanted cells derived from syngeneic iPSCs were not rejected after transplantation (56). Also, Araki R. et al., compared the immunogenicity of skin and bone marrow cells derived from mouse iPSCs to the immunogenicity of ESC-derived tissue and did not observe any differences between the two groups, finding limited immunogenicity in both cases (57). This support the idea that autologous iPSCs could be applied for cell replacement therapies without eliciting immune rejection. However, and revealingly, in the same study it was shown that cardiomyocytes derived from these same iPSCs elicited an immunogenic response, as observed by increased T-cell infiltration (57). On the other hand, Morizane et. al., found that autologous transplantation of iPSC-derived cells generated a minimal immune response compared with allografts in non-human primate brains in the absence of immunosuppression (58). They suggested that immunosupression was not necessary for autologous transplantation of iPSC-derived neural cells in the brain. In contrast, Liu et. al., differentiated iPSCs derived from human umbilical cord mesenchymal stem cells (UMCs) or skin fibroblasts (SFs) into neural progenitor cells (NPCs) and analyzed their immunogenicity. They reported a lower immunogenicity of NPCs differentiated from iPSCs derived from UMCs than from SFs (59), retaining a low immunogenicity as the parental UMCs. Hence, the authors suggested that the lower immunogenicity of UMCs could persist after cell reprogramming and further differentiation. This discovery goes in the line with the AGIIR hypothesis: that generation of functional lineages with lower immunogenicity from iPSCs strongly depends on genomic and epigenetic stability.

It is unclear whether iPSC-derived cells can be immunogenic at different extents as a consequence of aberrations acquired, and if the genetic alterations may affect or not the transplantation potential. Thus, it appears to be of great relevance to estimate the immunogenicity of clinical valuable cells, as well as the tissue specific propensity to become immunogenic depending on the number and type of cumulated defects. Work from our group demonstrated abnormal toll like receptor 3 (TLR3) gene methylation and expression in iPSC-derived cells, suggesting dysregulated innate immune responses (60).

**Figure 2:**
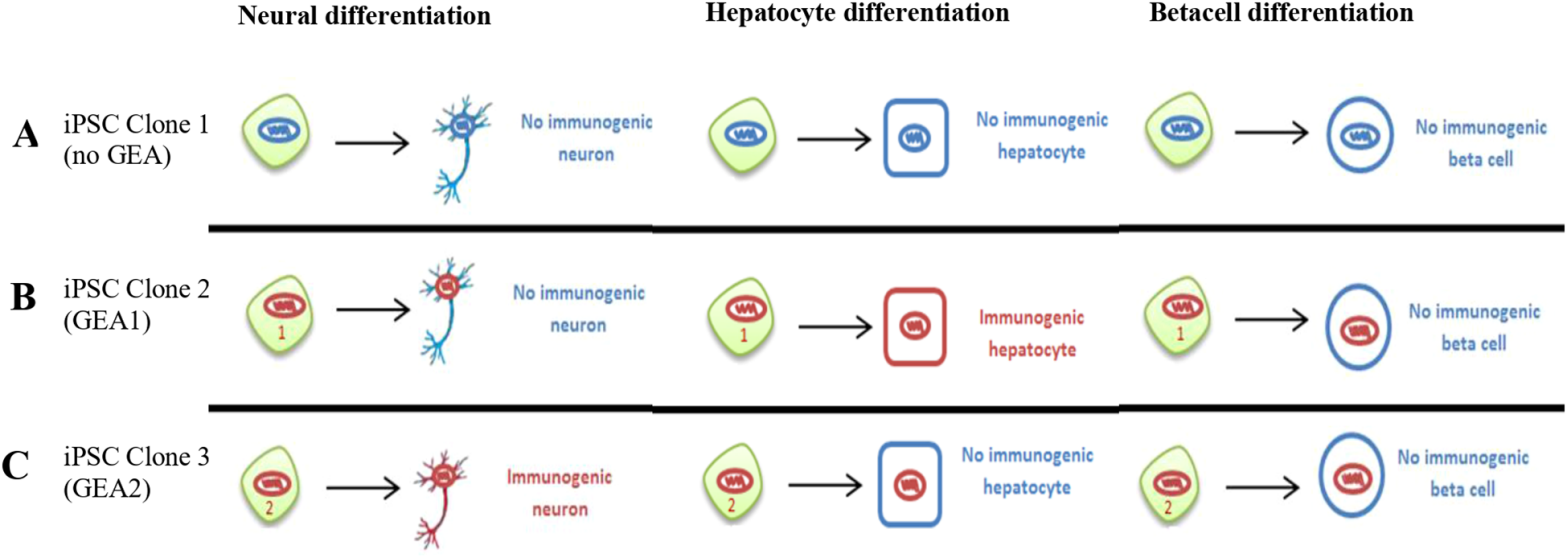
Hypothetical differentiation routes from three iPSC clones, with or without genetic and epigenetic aberrations. Clones not carrying genetic or epigenetic aberrations (GEA) are depicted with blue nucleus, and clones carrying aberrations with red nucleus. Human iPSCs differentiated into distinct cellular fates, where most of the derivatives are not immunogenic and are tolerated by the host. However, some derived cell types are immunogenic. **(A)**. Clone 1 carries no relevant genetic or epigenetic aberrations (no GEA). **(B, C)**. Clones 2 and 3 carry two different combinations of chromosomal aberrations: combinations 1 and 2. The hypothetical combination no1 of genetic and epigenetic aberrations (GEA1) found in clone 2 is one of the combinations that can originate immunogenic hepatocytes. On the other hand, the hypothetic combination n°2 of genetic and epigenetic aberrations (GEA2) found in clone 3 can produce non-immunogenic hepatocytes but produce immunogenic neurons. At the same time, all clones would be able to differentiate into non-immunogenic beta cells for instance. We propose to genetically and epigenetically characterize all iPSC clones derived from every patient to be able to identify and recognize the less immunogenic combination of genetic alterations.

Different immunogenic predispositions of iPSCs could depend on the cell type they are differentiated to (**Figure 2**). This idea was first proposed by Dr.’s Xu group (61), and was later reviewed (62).

## 3 Immune response of genetically unstable cells

In the race to the clinic, the potential immunogenicity of iPSC-derived cells has been largely overlooked. A number of publications have described mechanisms inferred from genetic and epigenetic instability to predict potentially elicit immune responses, and others have demonstrated *established* mechanisms observed in iPSC-derived cells, including our own work in 2019 on epigenetic changes in the toll-like 3 receptor (TLR3) (60) (**Table 1**).

**Table 1:** Summary of the five major mechanisms: proposed to underlie the immunogenicity of induced pluripotent stem cell (iPSC)-derived cells within the AGIIR hypothesis.

Mechanism	Description	Evidence status	Relevant references
Aberrant or ectopic expression of self-antigens.	Genetic or epigenetic dysregulation during reprogramming causes normally silent self-antigens in target cells to be expressed in iPSC-derived cells, may lead to presentation of self-peptides that might break tolerance and trigger T cell responses.	Hypothetical	57, 61–63
Overexpression of lineage-specific antigens.	Copy number changes or selective clonal expansion during culture lead to overexpression of tissue-specific proteins, potentially triggering immune recognition.	Hypothetical	16, 23
iPSC-specific neoantigens from reprogramming-associated mutations	Mutations acquired during reprogramming or early passages generate novel epitopes acting as neoantigens potentially recognized by the immune system.	Hypothetical	13, 41
Altered antigen processing or presentation pathways.	Reprogramming and differentiation affect molecules involved in antigen display (e.g., MHC-I and β2-microglobulin expression), altering the peptide repertoire presented to T cells.	Established	64, 65
Abnormalities in innate immune sensing.	Epigenetic changes affect innate immune pathways (e.g., TLR3) influencing cytokine signaling and immune activation.	Established	60

Each mechanism is classified as either established or hypothetical based on currently available evidence. Hypothetical: indicates mechanisms inferred from genetic and epigenetic instability and predicted to potentially elicit immune responses, whereas established indicates mechanisms experimentally observed in iPSC-derived cells. Evidence Status refers to if the mechanism has established data to support it or not.

As discussed above, the reprogramming process itself has a major impact on the genetic landscape that could impact autologous cell therapy approaches, suggesting that a potential immunogenic role of autologous cells transplantations could have been underestimated. More than a decade ago, it was proposed that genetic and epigenetic aberrations acquired during reprogramming could increase cell immunogenic potential (63).

The *altered genome induced immune response (AGIIR) hypothesis* postulates that cell reprogramming-derived genetic and epigenetic alterations may lead to immune dysregulation in certain cell types (**Figure 2**).

Some genomic alterations may affect genes or promoters involved in the differentiation of iPSCs to neurons but not to hepatocytes, provoking differentiation to immunogenic neurons but normal hepatocytes. Whether the most frequent alterations in pluripotent cells, like duplications on chromosomes 1, 12, 17 and X or the specific amplification in 20q, are destined to be immunogenic is yet to be determined. It seems likely that most iPSCs genomic aberrations are going to be harmless and only a few abnormalities will actually be hazardous. However, it is still an open question to know which kind of tissues differentiated from iPSCs can be immunogenic due to cell type-specific aberrations, such as cells abnormally expressing *Hormad1* and *Zg16* genes (61) or cells carrying other specific genetic and/or epigenetic aberrations still unknown. (61) demonstrated that after injecting retrovirally reprogrammed iPSCs in syngeneic recipients, induced T-cell-dependent immune response prevents the formation of teratomas in mice (61). Teratomas that did not regress were infiltrated with CD4^+^ T cells with apparent necrosis within parts of the tissue. This rejection was not observed after injection of syngeneic mouse ESCs (mESCs).

Regarding rejection of transplanted allogeneic cells and organs, the main immune response involves the major histocompatibility complex-I (MHC-I), expressed on every nucleated cell in the body, whose function is to present foreign antigens to T cells. Pick et al., demonstrated that during reprogramming, iPSCs downregulated expression of human leukocyte antigen (HLA)-A/B/C and β2 microglobulin (β2M) (64), the two components of MHC class I (MHC-I). Their results showed very low expression levels of MHC-I proteins on the surface of ESCs. During differentiation of ESCs, high levels of MHC-I expression are observed, resulting in an increase in immunogenicity in transplanted ESC-derived cells (65–67).

## 4 Conclusions and future perspectives

While human iPSC-derived cells immune response may appear minimal, immunogenicity may play a major role in their potential applicability in the clinic, as some subsets of genetic and epigenetic aberrations might have the potential to generate immune responses. The proposed *Altered Genome Induced Immune Response* (AGIIR) hypothesis might explain some of the immunogenic responses reported with iPSC-derived cells in the literature, such as T cell infiltration in teratomas (61) and immunogenic cardiomyocytes (57).

Finally, before clinical application of iPSCs derivatives becomes a reality, it is crucial to study the influence of these alterations accurately and assess the risk stratification for immunogenicity. Classifying the major genetic and epigenetic alterations that may elicit an immune response should be included as part of a standard operating procedures (SOPs) for the clinical use of human iPSCs.
